# Aerobic, resistance, and specialized exercise training in heart failure with preserved ejection fraction: A state-of-the-art review

**DOI:** 10.1007/s10741-025-10526-x

**Published:** 2025-05-15

**Authors:** Saeid Mirzai, Uttsav Sandesara, Mark J. Haykowsky, Peter H. Brubaker, Dalane W. Kitzman, Anthony E. Peters

**Affiliations:** 1 Section on Cardiovascular Medicine, Department of Internal Medicine, Wake Forest University School of Medicine, 1 Medical Center Blvd, Winston-Salem, NC 27101 USA; 2 Integrated Cardiovascular Exercise Physiology and Rehabilitation Lab, Faculty of Nursing, College of Health Sciences, University of Alberta, Edmonton, AB Canada; 3 Department of Health and Exercise Science, Wake Forest University, Winston Salem, NC USA; 4 Section on Gerontology and Geriatric Medicine, Department of Internal Medicine, Wake Forest University School of Medicine, Winston Salem, NC USA

**Keywords:** Cardiorespiratory fitness, 6-min walk distance, Quality of life, Exercise capacity, Physical function

## Abstract

Heart failure with preserved ejection fraction (HFpEF) is a growing public health burden, contributing to significant morbidity, mortality, and healthcare costs. Exercise intolerance, a hallmark of HFpEF, stems from central (cardiac and pulmonary) and peripheral (vascular and skeletal muscle) factors that result in reduced oxygen delivery and utilization by active muscles. With relatively few effective therapies, exercise training has emerged as a reliable and proven therapeutic intervention to improve exercise capacity and physical function in HFpEF. This review synthesizes evidence from the existing literature to describe and evaluate various exercise modalities in HFpEF. Moderate-intensity continuous training significantly improves peak oxygen consumption and symptom burden and is supported by a large evidence base in patients with HFpEF. High-intensity interval training has shown potential as an alternative regimen with particular benefit in highly selected populations. Multi-modality regimens and low-intensity training approaches are potentially suitable for patients with limited exercise tolerance or those who are more vulnerable or frail. The addition of resistance training may further improve muscle strength and functional capacity. Integrating exercise interventions with complementary dietary approaches has also shown potential for enhancing exercise capacity response. Lastly, emerging modalities, such as inspiratory muscle training and functional electrical stimulation, offer additional unique options. Despite robust evidence, challenges in the long-term durability of benefits, poor responder rates (~ 1/3 of participants), and implementation persist. Ongoing and future efforts can focus on evaluating long-term clinical outcomes (i.e., mortality and hospitalizations), developing more personalized exercise protocols, and applying sustainable implementation strategies in clinical practice.

**Supplementary Information:**

The online version contains supplementary material available at 10.1007/s10741-025-10526-x.

## Introduction

Heart failure with preserved ejection fraction (HFpEF) is a significant and growing public health challenge. It accounts for over half of all heart failure (HF) cases, particularly affecting older adults, women, African Americans, and individuals with comorbidities such as hypertension, obesity, and diabetes [1, 2]. Its prevalence continues to rise due to aging populations and the increasing burden of cardiometabolic diseases [2]. HFpEF is associated with high morbidity, frequent hospitalizations, and substantial healthcare costs [1]. Once hospitalized, mortality rates for HFpEF are comparable to those observed in HF with reduced ejection fraction (HFrEF) [1]. Given these poor outcomes, optimizing treatment requires leveraging all available therapeutic options, including pharmacologic and non-pharmacologic interventions.

**Fig. 1:**
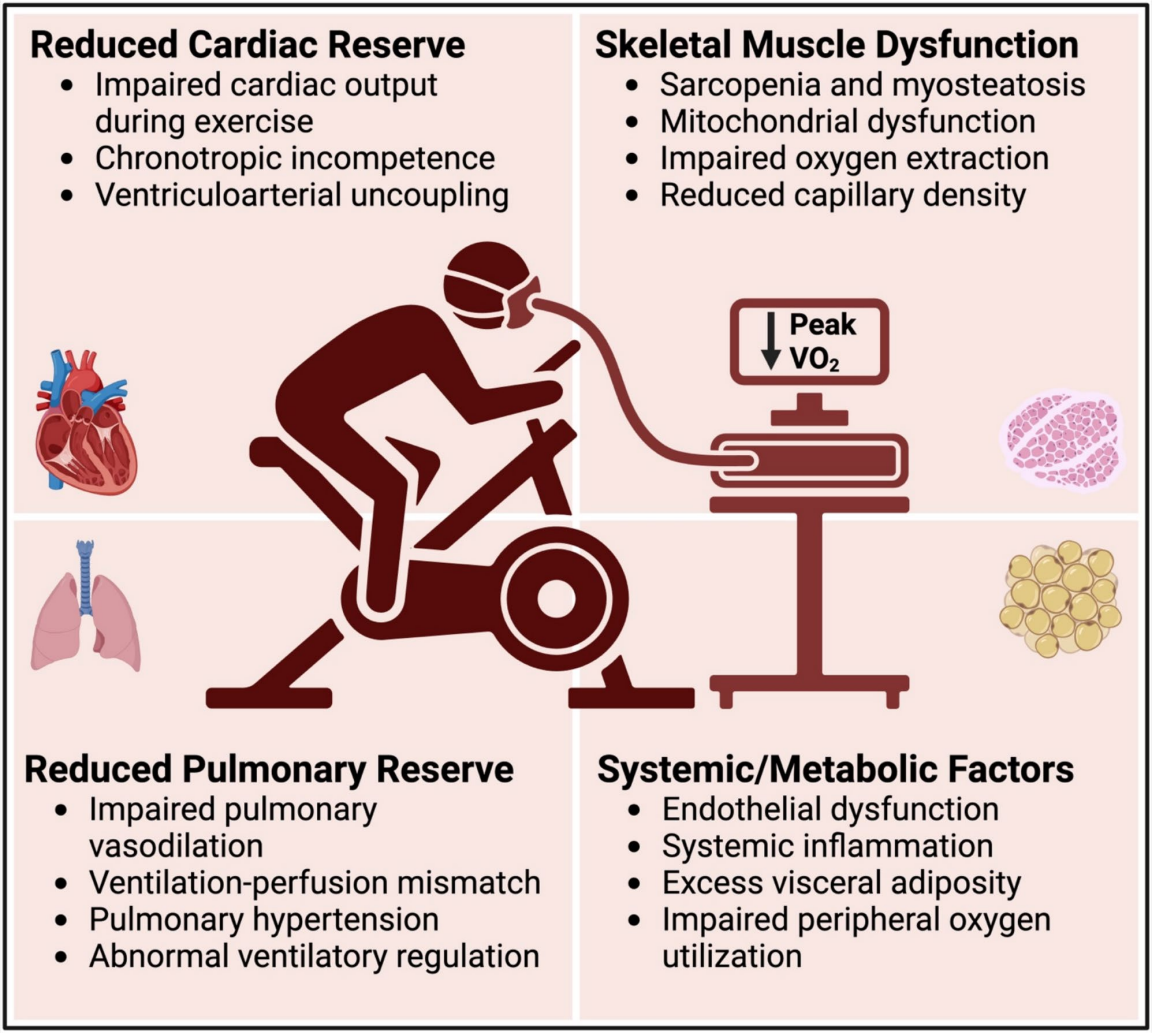
Contributors to exercise intolerance in heart failure with preserved ejection fraction

Exercise intolerance is a cardinal feature of HFpEF, significantly impairing quality of life (QoL) and functional independence, with peak oxygen consumption (peak VO_2_) approximately 30% lower than healthy controls [3]. Unlike HFrEF, where systolic dysfunction predominates, the mechanisms of exercise intolerance in HFpEF are multifactorial, encompassing central and peripheral contributors (Fig. 1). Cardiac factors include impaired diastolic filling, increased left atrial pressures, and limited cardiac output augmentation during exercise [4, 5]. Peripheral contributors, such as skeletal muscle dysfunction, reduced capillary density, and altered mitochondrial metabolism, exacerbate exercise limitations [4, 5]. Systemic inflammation and metabolic stress further compound these deficits, reducing exercise capacity [4, 5]. Most pharmacologic therapies have shown an absent or modest impact on exercise capacity in patients with HFpEF [6, 7]. While sodium-glucose cotransporter 2 (SGLT2) inhibitors show some promise in improving exercise capacity [8], exercise training shows substantial and robust benefits in HFpEF across numerous studies (Tables 1 and 2). Studies have also demonstrated significantly greater improvements in exercise capacity with training in HFpEF compared to HFrEF, achieved primarily through enhanced skeletal muscle function and peripheral oxygen utilization rather than central cardiac changes [3–5, 9, 10].

**Table 1:** Comprehensive summary of randomized trials evaluating exercise interventions in heart failure with preserved ejection fraction

Year	First author	Exercise participants (randomized/analyzed)	Control participants (randomized/analyzed)	Exercise group intervention	Control group intervention	Freq. (days/wk)	Duration (weeks)	Testing/analysis blinded	Key findings
2004	Gary [14]	16/15	16/13	Low-intensity walking, progression from 40 to 60% target HR	Education-only program, weekly home visits	3	12	Not described	Improved 6MWD, QoL, and depression scores
2010	Kitzman (PARIS 1) [15]	26/24	27/22	Supervised aerobic exercise (walking, cycling, 40–70% HRR)	Biweekly phone follow-up	3	16	Yes	Improved exercise capacity metrics and trends for QoL
2011	Edelmann (Ex-DHF Pilot) [16]	46/44	21/20	Combined endurance & resistance training, cycling (50–70% pVO_2_) and 60–65% 1RM resistance exercises	Usual care	2–3	12	Yes	Improved pVO_2_, QoL, diastolic function, and left atrial size
2012	Alves [17]	20/20	11/11	Aerobic interval training (cycling or treadmill, 70–75% HR max intervals with active recovery)	Usual care	3	24	Yes	Improved fitness (METs) and diastolic function
2012	Smart [18]	16/12	14/14	Supervised cycling at 60 RPM, 60–70% pVO_2_	Usual activity	3	16	Echo outcome blinded	Improved pVO_2_ but did not significantly affect diastolic function or QoL
2013	Karavidas [19]	15/15	15/15	FES of lower limb muscles	Placebo stimulation without muscle contractions	5	6	Yes	Improved endothelial function, exercise capacity (6MWD), QoL, and depression
2013	Kitzman (PARIS 2) [20]	32/24	31/30	Supervised walking/cycling, progressively increased intensity (40–70% HRR)	Biweekly phone-based retention follow-up	3	16	Yes	Improved pVO_2_ but did not improve endothelial function or arterial stiffness
2013	Yeh [21]	8/8	8/8	5 simplified tai chi movements adapted from Yang-style	Low-impact aerobic exercise class	2	12	Yes	Similar exercise capacity and QoL scores between groups
2014	Palau [22]	14/14	13/12	IMT using threshold inspiratory muscle trainer	Standard care	7	12	Yes	Improved pVO_2_, ventilatory efficiency, and QoL; no change in diastolic function
2015	Angadi [23]	9/9 (HIIT), 6/6 (MICT)	NA	HIIT: 4 × 4-min intervals at 85–90% HR peak, MICT: 30 min at 70% HR peak	NA	3	4	Yes	HIIT improved aerobic capacity and diastolic function; MICT showed no changes
2016	Fu* [24]	30/30	30/29	HIIT (alternating 3-min 40% and 80% pVO_2_ intervals)	General health care	3	12	Echo outcome blinded	Improved pVO_2_, non-invasive cardiac hemodynamics, and O_2_ delivery/utilization
2016	Kitzman (SECRET 1) [25]	26/24 (ET), 24/24 (diet), 25/22 (combined)	25/22	Supervised aerobic training ± caloric restriction with hypocaloric diet	Attention control	3	20	Yes	Improved pVO_2_; combined exercise + diet yielded additive pVO_2_ effects; no significant QoL changes
2017	Shaltout [26]	10/10	9/9	Supervised aerobic training with high-nitrate BRJ supplementation	Same exercise training with placebo	3	4	Yes	Improved pVO_2_ and blood pressure in both groups; BRJ provided no additional benefit
2018	Lang (REACH-HF) [27]	25/22	25/23	Home-based rehabilitation program (self-management manual, facilitator support)	Usual care	Individualized	12	Yes	Home-based rehab feasible with good adherence; trend toward improved QoL
2019	Palau (TRAINING-HF) [28]	15/13 (IMT), 15/13 (FES), 16/13 (IMT + FES)	13/13	IMT: Threshold inspiratory muscle training; FES: Lower limb electrical stimulation; IMT + FES	Usual care	IMT-BID; FES-2x/wk;	12	Yes	IMT, FES, and IMT + FES improved pVO_2_ and QoL, with effects sustained at 24 weeks
2020	Azhar [29]	8/5 (Protein + ET), 7/6 (Protein only)	8/6 (control)	35-min gym session 1x/week + 35-min water-based exercise 2x/week + protein supplementation (1.2 g/kg/day)	Monitored weekly without feedback	3	12	Not described	Protein + exercise improved 6MWD and blood pressure; protein alone showed no benefit
2020	Donelli da Silveira [30]	12/10 (HIIT), 12/9 (MICT)	NA	HIIT: 4 × 4-min intervals (80–90% pVO_2_); MICT: 47 min at 50–60% pVO_2_	NA	3	12	Yes	HIIT had greater improvements in pVO_2_ compared to MICT; both improved ventilatory efficiency, diastolic function, and QoL
2020	Kinugasa [31]	8/8	12/12	Home-based inspiratory muscle training (30% of max inspiratory muscle pressure)	Usual care	7	24	Not described	Improved inspiratory muscle strength and aerobic threshold; neutral pVO_2_
2021	Kitzman (REHAB-HF)* [32, 33]	93/93	92/92	Progressive multi-domain rehabilitation (balance, strength, mobility, endurance)	Usual care + attention control (biweekly calls)	3	12	Yes	Improved physical function (SPPB), 6MWD, and QoL; trend toward reduced hosp. and mortality
2021	Mueller (OptimEx-Clin) [34]	60/58 (HIIT), 60/58 (MICT)	60/60	HIIT: 4 × 4-min intervals (80–90% HRR); MICT: 5 × 40 min/week (35–50% HRR)	One-time guideline-based activity advice	HIIT: 3x/wk; MICT: 5x/wk	48 (12 supervised, 36 telem.)	Yes	Improved pVO_2_ for HIIT and MCT; no significant difference in pVO_2_ change between HIIT and MICT
2022	Alonso (HEART camp) [35]	25/21	34/31	Multicomponent behavioral program (HEART Camp) with aerobic (MICT) and resistance training	Paid facility access (enhanced usual care)	NR	72	Not described	Improved adherence, functional capacity (6MWD), health status, and symptoms
2023	Brubaker (SECRET 2) [36]	44/39 (RT + CR + AT), 44/38 (CR + AT)	NA	RT: 2 upper + 4 lower body exercises; AT: aerobic training; CR: hypocaloric diet	NA	3	20	Yes	Improved pVO_2_ and QoL in both groups; adding RT improved leg strength but did not further increase pVO_2_ or QoL
2023	Liu [37]	20/18 (ET + pill), 20/17 (ET only)	20/20	Exercise ± Shexiang Baoxin Pill (aerobic + resistance)	Conventional drug therapy only	5–7 (aerobic), 3 (RT)	12	Yes	ET improved fitness, sleep, and QoL; Shexiang Baoxin Pill provided additional benefits for sleep and QoL
2024	Borlaug (INABLE-training) [38]	55/37 (ET + nitrite), 36/36 (ET)	NA	Structured exercise (guidelines-based) + inorganic nitrite 40 mg or placebo 3 × daily	NA	3	12	Yes	Exercise improved pVO_2_ and QoL; nitrite did not enhance the effects of exercise
2024	Obaya [39]	24/20 (lower limb), 24/20 (upper limb)	NA	Lower limb aerobic exercise (cycling); upper limb aerobic exercise (arm ergometer)	NA	3	12	Not described	Improved pVO_2_ in both groups; higher increase in lower limb exercise
2024	Sharif [40]	12/9	12/10	Aerobic warm-up, RT: major muscle group exercises at 60% 1RM, core strengthening	Education and walking recommendation (30 min/day, 3x/wk)	3	12.5 ± 3.0	Not described	RT improved locomotor muscle composition and increased peak exercise workload, muscle strength, and muscle quality
2025	Edelmann (Ex-DHF) [41]	161/139	161/148	Combined endurance & resistance training: cycling (50–70% peak VO_2_) + resistance (60% 1RM)	Usual care	3	48	Yes	Improved peak VO_2_ and NYHA class; no change in diastolic function, QoL, or clinical outcomes

Abbreviations:* 1RM* 1 repetition maximum, *6MWD* six-minute walk distance, *AT* aerobic training, *BRJ* beetroot juice, *CR* caloric restriction, *EF* ejection fraction, *ET* exercise training, *FES* functional electrical stimulation, *HFpEF* heart failure with preserved ejection fraction, *HIIT* high-intensity interval training, *HR* heart rate, *HRR* heart rate reserve, *IMT* inspiratory muscle training, *MICT* moderate-intensity continuous training, *NA* not applicable, *NR* not reported, *QoL* quality of life, *RM* repetition maximum, *RT* resistance training, *pVO*_2_ peak oxygen consumption

^*^Data from HFpEF subset; HFrEF excluded

**Table 2:** Distribution and key findings of exercise modalities in heart failure with preserved ejection fraction clinical trials

Exercise modality	# of trials	# of participants*	Aggregate key findings
Moderate-intensity continuous	9	46 + 26 + 54 + 92 + 19 + 11 + 118_€_ + 67_Ω_ + 40 = **473**	Improved pVO_2_ Variable QoL findings
[15, 18, 20, 25, 26, 29, 34, 36, 39]			
Endurance + resistance _¥_	4	64 + 39_Ω_ + 73 + 287 = **463**	Improved pVO_2_, QoL Variable diastolic function findings
[16, 36, 38, 41]			
High-intensity interval	4	15_**_ + 30 + 19_β_ + 120_€_ = **184**	Improved pVO_2_ Variable diastolic function findings
[23, 24, 30, 34]			
Multi-domain physical rehabilitation _∞_	1	**185**	Improved SPPB, 6MWD, QoL
[32, 33]			
Inspiratory muscle training _£_	2	26 + 39_α_ = **65**	Improved pVO_2_, QoL
[22, 28]			
Functional electrical stimulation	2	30 + 39_α_ = **69**	Improved exercise capacity (pVO_2_ *or* 6MWD), QoL
[19, 28]			
Moderate-intensity interval	1	**31**	Improved fitness (METs), diastolic function
[17]			
Low-moderate intensity _π_	1	**28**	Improved 6MWD, QoL
[14]			

Abbreviations:* pVO*_2_ peak oxygen uptake, *QoL* quality of life, *6MWD* 6-min walk distance, *METs* metabolic equivalents, *MICT* moderate-intensity continuous training, *HIIT* High-intensity interval training, *IMT* inspiratory muscle training, *FES* functional electrical stimulation

^*^ Intervention + Control

^**^ Angadi et al. utilized MICT as a comparator group (*n* = 6) to HIIT and found no change in pVO_2_ with MICT; this total study count (*N* = 15) is included only in the HIIT row

_α_ Included IMT + FES combination group in both rows

_β_ Donelli et al. utilized MICT as a comparator group (*n* = 9) to HIIT and found improvement in pVO_2_ with MICT as well; this total study count (*N* = 19) is included only in the HIIT row

_∞_ REHAB-HF intervention

_£_ Home-based once-daily IMT (Kinugasa et al. 2020) not included

_€_ Control group (*n* = 60) for OptimEx-Clin included in both MCT and HIIT rows, along with respective intervention groups

_Ω_ With caloric restriction (two groups from Secret 2)

_¥_ Home programs: HEART camp and Liu et al. not included; Sharif et al. not included

_π_ Home program: REACH-HFpEF not included

Current guidelines from professional societies such as the American Heart Association (AHA), American College of Cardiology (ACC), Heart Failure Society of America (HFSA), and the European Society of Cardiology emphasize the importance of physical activity in managing HFpEF [3, 11, 12]. Exercise training (or regular physical activity/“exercise”) is recommended as a class I intervention in all patients with HFpEF who can participate safely to improve exercise capacity and QoL [11, 12]. These recommendations are grounded in a large body of evidence demonstrating that exercise training improves functional status, exercise performance, and QoL in patients with HFpEF [11, 12]. However, implementation challenges have made translating these findings into standardized clinical practice difficult, namely the lack of reimbursement by the Centers for Medicare & Medicaid Services [13].

This comprehensive review examines the substantial evidence supporting exercise training as a key therapeutic intervention for patients with HFpEF, exploring various exercise modalities, their physiological effects, and implementation strategies to improve patient outcomes.

## Methods

This review is based on a systematic literature search using MEDLINE/PubMed, Embase, and Cochrane databases from inception until January 28, 2025. Our search strategy combined: ("heart failure ADJ2 preserved ejection fraction"or"diastolic heart failure") AND ("exercise*"or"training") AND ("trial*"or"intervention*"). The proximity operator ADJ2 specifies that two terms must appear within two words of each other in any order. The asterisk (*) was used to capture variations of the terms (e.g., exercise, exercises, exercising). We included randomized controlled trials evaluating exercise interventions in patients with HFpEF. Conference abstracts and non-English studies were excluded. Additionally, we performed citation searching of included articles to identify other relevant studies.

The search yielded 2,122 potentially relevant articles. After screening titles, abstracts, and full texts according to our inclusion criteria, 21 trials were selected. An additional 6 trials were identified through citation searching, resulting in 27 included studies (Supplemental Figure S1). These studies assessed various exercise modalities and their effects on this population’s exercise capacity, QoL, and physiological outcomes. The evidence from these trials and meta-analyses using them served as the foundation for this review.

## Overview of exercise training protocols in HFpEF

Exercise training protocols for patients with HFpEF encompass a variety of structured regimens that address distinct physiologic impairments associated with the condition. These include aerobic training modalities, which entail moderate-intensity continuous training (MICT), high-intensity interval training (HIIT), and low-intensity training (LIT), as well as resistance training and combined protocols. These different approaches may be selected based on the individual patient’s capacity, clinical status, and therapeutic goals.

Individual study characteristics, including exercise intervention details and outcomes, are summarized in Table 1 and Supplemental Table S1 [14–41]. MICT typically involves steady-state aerobic exercise at 50–70% of heart rate reserve (HRR = maximum heart rate—resting heart rate) or peak VO_2_, often walking or cycling [15, 18, 20, 25, 26, 29, 34, 36, 39]. HIIT consists of interval training with periods of high-intensity exercise at 80–90% of peak heart rate or VO_2_, interspersed with active recovery periods at 40–50% [23, 24, 30, 34]. LIT emphasizes light physical activity, such as walking, at 40% of heart rate reserve or peak VO_2_, progressing to longer durations as tolerated [14]. Resistance training in HFpEF trials has involved strengthening exercises, though the specific intensity and repetition parameters vary across studies [16, 36, 37, 40, 42–44]. Combined training may integrate aerobic exercise with resistance training and, in some cases, a hypocaloric diet for weight loss to address the cardiovascular, musculoskeletal, and metabolic limitations of HFpEF [16, 25, 36, 37, 43–45].

## Outcomes by exercise modality

### Aerobic training

Clinical trials and associated meta-analyses consistently demonstrate that aerobic exercise training enhances exercise capacity in patients with HFpEF. These interventions, performed at varying intensities, significantly increase peak VO_2_, a vital marker of cardiovascular fitness and prognosis in HF [20, 46–48]. The improvements typically exceed the clinically meaningful threshold of ~ 1.0 mL/kg/min (6–7%) [49, 50]. Patients also show meaningful functional gains through increased six-minute walk distance (6MWD) and enhanced QoL measures [20, 46–48]. While traditional protocols have centered on MICT using structured walking or cycling, recent research has expanded to include HIIT, which combines aerobic and anaerobic stimuli, and LIT approaches.

### Moderate-intensity continuous training

Moderate-intensity continuous training represents a well-established approach to aerobic training in HFpEF. Meta-analyses have demonstrated robust, statistically significant improvements in exercise capacity, with peak VO_2_ increases of 2.05 mL/kg/min (95% CI 0.81 to 3.29) compared to controls [15, 18, 34, 51]. This finding is strongly supported by many studies (Tables 1 and 2), including SECRET 1 (N = 100), SECRET 2 (N = 88), and OptimEx-Clin (N = 180) trials [34]. In the OptimEx-Clin trial specifically, while the study’s predetermined target of 2.5 mL/kg/min improvement in peak VO_2_ set an especially high bar that was not achieved, meaningful benefits were demonstrated, with 3 months of MICT achieving substantial improvements in peak VO_2_ (1.6 mL/kg/min) compared with controls (−0.6 mL/kg/min) [34, 52]. Beyond cardiorespiratory fitness, MICT has shown beneficial effects on QoL, with Minnesota Living with Heart Failure Questionnaire (MLHFQ) scores decreasing by 8.4 points (95% CI −17.6 to 0.9) on pooled analyses [15, 18, 51]. Additionally, the 6MWD increased by 35.7 m (95% CI −63.0 to 134.4), exceeding the clinically meaningful threshold of 32 m for patients with HF, though not reaching statistical significance [15, 51].

### High-intensity interval training

High-intensity interval training combines aerobic and anaerobic energy systems, with research showing that most of the energy during intermittent exercise is derived from aerobic metabolism [53]. It has recently emerged as a potential exercise modality for patients with HFpEF. Early studies suggest potential benefits, though the evidence base is still developing. Meta-analyses indicate HIIT achieves peak VO_2_ improvements of 2.88 mL/kg/min (95% CI 1.36 to 4.39) compared to controls [34, 51]. A similar magnitude of benefit has been noted in other meta-analyses, with one study showing peak VO_2_ gains of 3.5 mL/kg/min (95% CI 2.6 to 4.4) compared to controls [23, 30, 48]. Quality of life measures also demonstrate improvements, with HIIT reducing MLHFQ scores by −14.45 points (95% CI −24.81 to −4.10), nearly triple the clinically meaningful threshold [24, 51]. However, given the physically demanding nature of HIIT, careful patient selection and monitoring are essential.

### Moderate-intensity continuous versus high-intensity interval training

Direct comparisons between MICT and HIIT have provided important insights regarding their relative efficacy in HFpEF. A meta-analysis of three trials found no significant differences in peak VO_2_ between modalities [23, 30, 34, 54]. The OptimEx-Clin trial also showed similar improvements in peak VO_2_ between MICT and HIIT over 3 and 12 months [34]. However, completion rates for HIIT declined from 80% at 3 months to less than 60% at 12 months, suggesting potential challenges with long-term adherence. A secondary analysis of the trial found that exercise frequency and duration were more strongly associated with improvements in peak VO_2_ than exercise intensity in both HIIT and MICT groups [13]. Both modalities showed equivalent benefits when adjusted for energy expenditure [13]. Thus, while both approaches appear effective, MICT’s established track record, potentially better tolerability and long-term adherence, and larger safety database may make it preferable for many patients with HFpEF, with emphasis placed on maintaining regular exercise frequency and duration rather than intensity [50].

### Low-intensity training

Studies examining LIT in HFpEF are limited, though this approach may be particularly valuable for deconditioned patients or those with significant symptom burdens. In one of the few trials utilizing this method, a 12-week home-based walking program starting at 40% intensity and progressing to 60% significantly improved 6MWD compared to the education-only control group (between-group *p* = 0.002) [14]. Quality of life also improved significantly in the intervention group as measured by the MLHFQ (between-group *p* < 0.014) [14]. This study included older women with multiple comorbidities, demonstrating that LIT can be effectively implemented in more vulnerable populations who might find higher-intensity protocols too demanding. The gentler approach of LIT may allow patients to begin and maintain an exercise program with lower dropout risk, potentially leading to better long-term adherence. This sustained participation could prove more beneficial than achieving higher intensities but risking program discontinuation, and LIT may serve as a foundation for gradually progressing to higher intensities as fitness improves.

### Resistance training

Recent studies examining resistance training in patients with HFpEF have demonstrated meaningful benefits, though data remains limited. In one of the few available trials, both resistance training and control groups showed improvements in peak VO_2_, but resistance training provided additional advantages in peak workload (resistance training: 98 ± 32 to 113 ± 28 vs. control: 110 ± 19 to 108 ± 25 workload, interaction *p* < 0.05) and exercise duration (resistance training: 19.2 ± 5.0 to 21.6 ± 4.3 vs. control: 20.7 ± 3.2 to 20.9 ± 3.7 min, interaction *p* < 0.05) [40]. Building on these benefits, although protein supplementation alone (1.2 g/kg/day) has not benefited patients with HFpEF [29], an ongoing trial is investigating its potential synergistic effects when combined with resistance training [55], which could help optimize rehabilitation protocols. Compared with aerobic training, resistance training has produced comparable improvements in functional capacity, with both modalities showing similar enhancements in peak VO_2_ [56]. These interventions proved safe and feasible, with > 80% compliance and no adverse events reported [40]. While these initial findings suggest that resistance training is an effective exercise modality for improving exercise tolerance in patients with HFpEF, larger trials are needed to definitively evaluate its role in HFpEF management.

When combined with aerobic training, resistance protocols have not consistently demonstrated additive benefits beyond those achieved with either modality alone. Meta-analyses reveal varying improvements in aerobic capacity vs. controls: combination of MICT and resistance training increased peak VO_2_ by 1.85 mL/kg/min (95% CI 0.27 to 3.44) vs. 2.05 mL/kg/min (95% CI 0.81 to 3.29) with MICT alone [16, 51, 57], while combined exercise improved peak VO_2_ by 3.2 mL/kg/min (95% CI 1.4 to 5.0) vs. 3.5 mL/kg/min (95% CI 2.6 to 4.4) with HIIT alone [16, 42, 48]. Regarding QoL and functional capacity, combined aerobic and resistance exercise showed modest improvements below clinically meaningful thresholds, with MLHFQ scores decreasing by −3.00 points (95% CI −14.50 to 8.50) and 6MWD increasing by 7.00 m (95% CI −84.61 to 98.61) [16, 51]. While more robust trials are needed, one notable study examining resistance training added to aerobic training plus caloric restriction found that although both groups achieved similar peak VO_2_ improvements, the resistance training group showed greater gains in leg strength (+ 4.9 [95% CI 0.7 to 9.0] vs. −1.1 [95% CI −5.5 to 3.2] newton meter [Nm], interaction *p* = 0.05) and muscle quality (+ 0.07 [95% CI 0.03 to 0.11] vs. + 0.02 [95% CI −0.02 to 0.06] Nm/cm^2^, interaction *p* = 0.04), despite comparable losses in skeletal muscle mass from weight loss [36].

Most recently, combined endurance and resistance training was evaluated in the largest randomized exercise trial in patients with HFpEF [41]. In the Ex-DHF study, 322 stable patients with HFpEF were randomized to either 12 months of combined training or usual care. While the primary composite endpoint (modified Packer score) did not demonstrate significant improvement with exercise training compared to usual care, analysis of secondary outcomes revealed clinically meaningful benefits in peak VO_2_ (mean difference 1.3 ml/kg/min, 95% CI 0.4–2.1) and NYHA functional class (odds ratio 7.77, 95% CI 3.73–16.21) [41]. The intervention was found to be safe; however, a notable challenge was the relatively low adherence rate, with only 38.1% of patients completing at least two supervised sessions per week over the 12 months [41]. These findings suggest that while combined endurance and resistance training can improve important clinical parameters in patients with HFpEF, maintaining long-term adherence remains a significant barrier to optimizing outcomes.

### Combined protocols

#### Aerobic training with dietary interventions

Combining aerobic training with dietary modifications, particularly hypocaloric diets, has shown promising results. Low-intensity exercise combined with a hypocaloric diet has demonstrated meaningful improvements in exercise capacity, with an observed increase in peak VO_2_ of 2.37 mL/kg/min (95% CI 1.02 to 3.71) compared to controls [25, 51, 58]. This combination was also effective for improving functional capacity, showing large increases in the 6MWD with a mean improvement of 80.46 m (95% CI −12.83 to 173.76) compared to controls [25, 51]. Dietary interventions typically involved structured meal plans with specific caloric targets, with protocols that may involve prepared meals with prescribed calorie intake deficits of approximately 400 kcal/day for diet-only groups and approximately 350 kcal/day for exercise-plus-diet groups [25, 51]. The safety profile of combined protocols remains favorable when interventions are appropriately tailored to individual capacity and monitored closely, and the evidence suggests that these combined approaches may offer a more comprehensive treatment strategy for patients with HFpEF, addressing both functional capacity and metabolic aspects of the condition.

#### Multi-domain rehabilitation

The REHAB-HF trial addressed a critical yet previously overlooked population: older adults hospitalized with acute decompensated HF [32]. This population differs markedly from the stable outpatients studied in prior exercise trials, as patients with acute decompensated HF exhibit severe deficits across multiple functional domains, including balance and mobility impairments that may increase falls and injuries with traditional exercise. Recognizing these unique challenges, REHAB-HF developed a novel, comprehensive, tailored, and progressive program targeting four physical function domains: strength, balance, mobility, and endurance [32]. The intervention began during or shortly after hospitalization and transitioned to outpatient sessions, with 60-min sessions conducted three times weekly for 12 weeks [32]. In the primary REHAB-HF trial of 349 older adults hospitalized with acute decompensated HF, there were large, statistically significant improvements in physical function with the intervention, with a mean between-group difference in Short Physical Performance Battery (SPPB) score of 1.5 points (95% CI 0.9 to 2.0, *p* < 0.001) [32]. A secondary analysis focusing on patients with HFpEF (*n* = 185) revealed they had significantly worse baseline physical function, frailty, QoL, and depression compared to patients with HFrEF [33]. Although interaction testing for 3-month functional outcomes was not statistically significant, the magnitude of improvement appeared larger in patients with HFpEF vs. HFrEF for several key measures, including SPPB, 6MWD, and QoL [33]. Moreover, patients with HFpEF showed greater potential benefits in clinical outcomes, with a nominally lower rehospitalization rate and significantly greater treatment benefits for all-cause death (interaction *p* = 0.080) and the global rank endpoint (interaction *p* = 0.098) [33]. These promising findings, specifically in patients with HFpEF, have influenced the design of the ongoing REHAB-HFpEF trial to determine whether this novel rehabilitation intervention can improve clinical outcomes in this frail, high-risk older population hospitalized with acute decompensated HFpEF [59].

### Specialized exercise modalities

#### Inspiratory muscle training

Inspiratory muscle training (IMT) targets respiratory muscle weakness, a key contributor to dyspnea and exercise intolerance in HFpEF [60]. Meta-analyses consistently demonstrate that IMT significantly improves cardiorespiratory fitness, with three independent studies reporting substantial increases in peak VO_2_: 2.72 mL/kg/min (95% CI 1.44 to 3.99, *p* < 0.001) [22, 28, 61], 2.82 mL/kg/min (95% CI 1.90 to 3.74, *p* < 0.001) [22, 31, 62–64], and 3.6 mL/kg/min (95% CI 2.3 to 4.9, *p* < 0.001) [22, 28, 48]. Through 24 weeks of continuous home-based training, IMT demonstrated sustained improvements in exercise capacity (peak VO_2_ increase 2.18 mL/kg/min, 95% CI 0.38 to 3.99, *p* < 0.001) [31, 62, 63]. The intervention also yields substantial functional capacity gains, with participants achieving increases in 6MWD of 83.97 m (95% CI 59.18 to 108.76, *p* < 0.001) [22, 62, 63]. Regimens typically include 20-min sessions performed twice daily, complemented by one supervised weekly session.

Beyond improvements in peak VO_2_, IMT offers additional physiologic and clinical benefits. The intervention enhances ventilatory efficiency, as demonstrated by reductions in the VE/VCO_2_ slope (−3.36, 95% CI −6.17 to −0.54, *p* = 0.019), achieved through improved respiratory muscle recruitment and delayed diaphragmatic fatigue [22, 28, 61]. Patients also experience meaningful improvements in QoL, with MLHFQ scores improving by more than 11 points (MD −11.49, 95% CI −20.08 to −2.91, *p* = 0.009) [22, 28, 61]. Given its safety profile, with no reported major adverse events, and low cost, IMT represents an effective option, either as a primary intervention or “bridge therapy,” particularly for patients facing barriers to conventional exercise participation [61, 62].

#### Emerging exercise modalities and delivery methods

Novel approaches to exercise intervention in HFpEF include functional electrical stimulation (FES). FES uses small electrical impulses delivered through electrodes placed on the skin to induce muscle contractions, effectively simulating exercise in targeted muscle groups. Small studies show that FES can improve peak VO_2_ (2.28 mL/kg/min, 95% CI 0.92 to 3.65, *p*** = **0.001), exercise capacity (increase in 6MWD by 52.77 m, 95% CI 30.61 to 74.93, *p* < 0.001), and QoL measures (reduced MLHFQ score by −14.74, 95% CI −22.44 to −7.08, *p* < 0.001) in patients with HFpEF [19, 28, 61]. This modality could serve as a “bridge therapy” to exercise training, particularly beneficial for patients unable to perform conventional exercise; however, these findings are based on two small trials, making it premature to draw definitive conclusions about FES’s role in HFpEF management despite the promising initial results [61].

Alternative exercise modalities, such as tai chi, have shown potential benefits for patients with HFpEF. In a randomized trial of tai chi compared to aerobic exercise (*N* = 16), 12 weeks of tai chi training resulted in a similar change in peak VO_2_, but there was a significantly greater improvement in 6MWD compared to the aerobic exercise group (69 ± 46 vs. 10 ± 31 m, *p* = 0.02) [21]. The groups also had similar improvements in MLHFQ scores, but depression scores improved more in the tai chi group (−1.7 ± 2.8 vs. 1.6 ± 3.0, *p* = 0.05) [21]. Notably, these benefits were achieved despite tai chi showing lower physiologic burden (i.e., oxygen uptake) during training sessions than aerobic exercise (4.3 vs 9.4 mL/kg/min, *p* < 0.01) [21].

Traditional dance has also emerged as a promising exercise modality for patients with HFpEF. A randomized trial with 51 male patients with HFpEF evaluated an 8-month Greek traditional dance program [65]. The dance group showed a significant improvement in peak VO_2_ (19.5 to 26.1 mL/kg/min, *p* < 0.05), comparable to improvements seen in the formal aerobic and resistance training group (19.5 to 25.8 mL/kg/min, *p* < 0.05) [65]. QoL indices improved significantly in both groups; however, only the dance group demonstrated a significant increase in intrinsic motivation scores (3.08 to 3.87 on a 5-point scale, *p* < 0.05) [65]. Adherence rates were high in both groups but significantly better in the dance group than in formal exercise (96.3% vs. 91.5%, *p* < 0.05) [65].

Finally, digital and home-based delivery of exercise rehabilitation has emerged as an effective alternative to traditional center-based programs for patients with HFrEF, accelerated by adaptations during the COVID-19 pandemic [66]. Meta-analyses of HF exercise trials show no significant differences between home-based vs. center-based delivery for mortality (interaction *p* ≥ 0.18), all-cause hospitalization (interaction *p* ≥ 0.51), or health-related QoL (interaction *p* ≥ 0.18), though center-based programs showed greater reductions in HF-related hospitalizations long-term (interaction *p* = 0.007) [66]. While these delivery methods may help address low cardiac rehabilitation uptake rates, evidence specific to patients with HFpEF remains limited, as most included trials focused on HFrEF.

## Mechanistic impacts of exercise

### Exercise training and cardiac effects

While aerobic exercise significantly improves exercise capacity, its impact on cardiac parameters is relatively modest. Studies show mixed results regarding diastolic function improvements, with some demonstrating reduced E/e’ ratios and enhanced diastolic function with HIIT [23, 24, 30], while others find no significant changes in E/e’, e’ velocity, or left atrial volume index [67, 68]. Notably, Mueller et al. found no differences between HIIT, MICT, and controls in diastolic parameters in a 12-month study [34]. No studies reported meaningful changes in cardiac chamber dimensions, systolic function, or cardiac output [15, 18]. This suggests that peripheral adaptations, rather than central cardiac changes, likely account for the improved exercise tolerance seen with aerobic training in patients with HFpEF. In contrast, emerging evidence suggests resistance training may have more pronounced effects on cardiac function, with one study demonstrating greater improvements in diastolic function through larger reductions in the E/e’ ratio and NT-proBNP levels [56]. However, these promising findings are based on limited data, and larger, well-designed trials are needed to confirm the potential cardiac benefits of resistance training.

### Exercise training and peripheral effects

Exercise training leads to significant peripheral adaptations in patients with HFpEF through different mechanisms depending on the type of training. For aerobic training, the primary adaptation for improved exercise capacity appears to be peripheral rather than central, including improved arteriovenous oxygen difference and enhanced oxygen extraction by exercising muscles [24, 68]; evidence from animal models also demonstrates improved mitochondrial function [69–71]. When resistance training is incorporated, there are additional beneficial peripheral adaptations. Studies show that resistance exercise leads to improved leg strength, enhanced muscle quality, and favorable changes in muscle composition, with significant improvements in leg lean percentage, decreases in leg fat percentage, and reductions in leg fat mass to lean mass ratio [36, 40]. These peripheral adaptations are particularly important given that patients with HFpEF exhibit abnormalities in both skeletal muscle quantity and quality, with a reduced percentage of lean mass and greater intramuscular adipose tissue that contribute to exercise intolerance [72–74].

## AHA/ACC/HFSA stage B heart failure

Understanding exercise interventions at earlier stages of the HF continuum could provide essential guidance for a comprehensive approach to HFpEF management. While most studies have focused on symptomatic, stage C HFpEF for exercise interventions, stage B HF, defined as structural heart disease without clinical signs/symptoms of HF, represents an important opportunity to pursue HFpEF prevention. In patients with left ventricular hypertrophy and elevated cardiac biomarkers (*N* = 46), one year of structured exercise training (HIIT plus strength training) increased peak VO_2_ by 21% compared to no change in controls (interaction *p* = 0.0004) [43]. The exercise group also demonstrated increased left ventricular end-diastolic volume (interaction *p* < 0.0001) and significant reductions in both LV chamber stiffness and myocardial stiffness (interaction *p* = 0.015 and *p* = 0.023, respectively) compared to controls [43]. In pharmacologically treated hypertensive patients with abnormal left ventricular relaxation and preserved ejection fraction (*N* = 88), a 6-month intervention focused on exercise and weight loss (combined cycle ergometer training with hypocaloric diet) showed significant improvements compared to controls in exercise duration and maximal exercise capacity (interaction *p* < 0.0001 for both), left atrial volume index (interaction *p* < 0.0001), and E/A ratio (interaction *p* < 0.0001) [45].

In patients with type 2 diabetes mellitus and diastolic dysfunction (*N* = 47), 12 weeks of HIIT was superior to MICT, with significant interaction effects for peak VO_2_ (interaction *p* = 0.002) and diastolic function parameters, including early filling velocity E (interaction *p* = 0.05) and e’ velocity (interaction *p* < 0.001) [75]. In another study (*N* = 223), a three-year exercise and lifestyle intervention combining initial gym-based training with telephone-guided home exercise did not significantly improve diastolic dysfunction or exercise capacity vs. controls in the intention-to-treat analysis [57]. However, among patients completing the full 3-year program, control group membership was independently associated with diastolic dysfunction (odds ratio 2.46, *p* = 0.034), suggesting benefits with sustained exercise adherence [57].

In summary, these data are encouraging for potential exercise training benefits in stage B HFpEF but require further exploration in larger studies and detailed delineation between undiagnosed, early-stage HFpEF and true, asymptomatic stage B HFpEF.

## Best practice guidance for exercise prescription in HFpEF

Best practices for exercise training “prescription” in HFpEF for optimal response rate and clinical outcomes remain an area of active research, but guidance can be drawn from the existing evidence base. For stable, compensated individuals with HFpEF in the outpatient setting, MICT is the most evidence-based, first-line therapy. Optimally, it is initiated under the supervision of an exercise physiologist or physical therapist in a controlled environment, consisting of 3–4 sessions per week of aerobic exertion (e.g., walking or biking), beginning with a warm-up followed by 1–2 intervals of 10–30 min at 30%−50% of HRR and titration up to standard MICT with 45–60 min sessions at 50–60% of HRR [76–78]. Patients should be reassessed every 3 weeks to ensure progression. Exercise intensity should be advanced as tolerated. Incorporating resistance/strength training should be strongly considered as tolerated and feasible [16, 36]. In select patients (i.e., relatively younger, less frail, or prior experience with exercise), HIIT can be considered an alternative initial regimen strategy to MICT. For older and deconditioned individuals with obesity, HIIT with shorter high-intensity intervals (approximately 1 min at 75–85% HRR with active recovery, as opposed to more standard HIIT 4-min intervals) has been proposed to support improved tolerance and adherence [77].

Alternative therapeutic interventions may be considered for patients with barriers to conventional exercise protocols, including IMT, FES, tai chi, and dance-based activities. Comprehensive exercise prescriptions must meticulously consider individual patient factors: clinical status, age, weight, musculoskeletal limitations, and personal preferences. The fundamental strategy emphasizes a gradual progression model prioritizing patient safety, incrementally building confidence and functional capacity, and potentially transitioning from supervised clinical sessions to sustainable home-based regimens [78]. Continuous patient monitoring and adaptable protocols are essential to maximize therapeutic potential, ensuring exercise interventions are dynamically tailored to each individual’s unique physiologic and psychologic profile.

## Special considerations and implementation

### Implementation challenges

Multiple barriers limit the adoption of exercise interventions in HFpEF (Table 3). At the patient level, physical limitations such as obesity, osteoarthritis, and reduced mobility, as well as symptoms of dyspnea, fatigue, and musculoskeletal pain, often challenge participation [14]. Psychological factors, including anxiety, depression, and fear of symptom exacerbation, may further discourage engagement [79]. A lack of awareness about exercise benefits, particularly its role in symptom relief and QoL improvement, contributes to suboptimal adherence [67]. Access-related obstacles represent a significant burden, as many patients encounter a combination of geographic distance from specialized facilities, inadequate transportation options, and limited local infrastructure [80, 81]. Financial constraints, including inconsistent insurance coverage, disproportionately affect patients in rural and underserved areas where these access barriers are often most pronounced [81].

**Table 3:** Barriers to adopting exercise interventions in heart failure with preserved ejection fraction and potential solutions

Barrier level	Specific barriers	Potential interventions and solutions
Patient barriers	Physical limitations: obesity, osteoarthritis, reduced mobility	Multi-modality interventions (i.e., REHAB-HF) Initiation with low-intensity therapies (LIT, FES, or IMT) Home-based programs with gradual intensity
	Psychological factors	Behavioral interventions, motivational interviewing
	Lack of awareness of benefits	Patient and provider education
Evidence base barriers	Unproven association with hospitalization/mortality	Outcomes trials
	Signals for low durability of effect	Additional formal evaluation of durability and investigation of durability strategies
	Non-/low-responder rates	Studies to define drivers of non-response and alternative strategies to target non-responders
Healthcare system barriers	Transportation issues	Telemedicine and mobile health
	Financial constraints	Policy advocacy for insurance coverage
	Lack of provider training	Provider training workshops, best practice statements

Abbreviations:* FES* functional electrical stimulation, *IMT* inspiratory muscle training, *LIT* low-intensity training

Ambiguity also persists regarding referral pathways to exercise programs. Structured exercise training programs involve supervised sessions with interventions tailored to enhance exercise capacity and QoL in patients with HFpEF [3]. Traditional cardiac rehabilitation programs, while supervised, are often designed for patients who are post-myocardial infarction or with HFrEF rather than optimized for the unique needs of patients with HFpEF [3]. Self-directed exercise approaches involve unsupervised activity and, to date, may lack the structure or intensity needed for long-term benefit in patients with HFpEF. A lack of study results dissemination and education among healthcare providers in prescribing exercise interventions may contribute to these programs'reduced utilization [82].

Further, despite numerous positive randomized trial results (Tables 1 and 2), heterogeneity in response to exercise training remains a significant challenge. For instance, post-hoc analyses of the PARIS trial revealed that 40% of participants were low responders (i.e., peak VO_2_ increase < 10%) [46]. Moreover, data on the durability of benefits is limited. The OptimEx trial demonstrated that the initial improvements in peak VO_2_ from HIIT and MICT diminished to non-significance between 3 and 12 months, coinciding with reduced exercise adherence during the home-based phase [34]. Similarly, the SECRET trial’s long-term follow-up showed that the positive impact of exercise diminished over time with weight regain post-intervention that was predominantly adipose tissue, leading to worsening body composition [83]. This underscores the need to identify predictors of response and implement strategies to extend and sustain benefits across diverse patient populations.

Addressing these challenges requires a multi-faceted and tailored approach to improve adherence [84]. Behavioral interventions, such as motivational interviewing and goal-setting frameworks, have shown promise in enhancing adherence [85]. Social support, including group-based sessions and caregiver involvement, fosters accountability and emotional reinforcement, making long-term engagement more feasible [81]. Home-based programs allow for gradual progression and intensity titration to improve functional performance and QoL without increasing the risk of adverse events, proving valuable for patients with frailty or mobility limitations who may struggle to attend traditional in-person sessions [14, 81, 86]. Telemedicine platforms and smartphone applications have emerged as practical tools for extending access to structured programs, particularly for patients with limited access to in-person services [87].

### Interaction with other therapies

Pharmacologic therapies targeting HFpEF pathophysiology can exert varying effects on exercise training. Diuretics effectively alleviate pulmonary congestion and dyspnea, improving the ability to engage in physical activity. However, chronic overuse may reduce preload and cardiac output, potentially impairing exercise tolerance [88]. Beta-blockers, often prescribed for managing comorbidities like atrial fibrillation (AF) and coronary artery disease (CAD), blunt the chronotropic response to exercise, limiting heart rate and cardiac output increases during activity, which can reduce exercise tolerance [89]. Careful dose adjustments may mitigate these effects, as some studies have suggested that beta-blocker withdrawal could improve exercise capacity and symptoms in select patients [90].

SGLT2 inhibitors have emerged as an important therapeutic option in HFpEF. Benefits include weight loss, improved metabolic efficiency, and cardiorenal protection through preload and afterload reduction via natriuresis and osmotic diuresis, alleviating symptoms like dyspnea and fatigue [91–93]. Their anti-inflammatory effects and ability to mitigate myocardial fibrosis enhance diastolic function [91–93]. Notably, the PRESERVED-HF trial demonstrated that SGLT2 inhibitor therapy significantly improved QoL and functional capacity, with clinically meaningful improvements in 6MWD and Kansas City Cardiomyopathy Questionnaire (KCCQ) scores [8]. Data regarding the impact of SGLT2 inhibitors on musculoskeletal quantity and quality are mixed [94]. Some studies have found skeletal muscle loss associated with SGLT2 inhibitor use [95], while others have suggested optimized muscle metabolism through enhanced mitochondrial function and other pathways [96, 97]. In this setting, SGLT2 inhibitors and exercise training interventions could complement each other when utilized together or in sequence (i.e., pharmacotherapy to stabilize HFpEF followed by structured exercise programs), but more research is needed [98].

More recently, several studies have highlighted the efficacy of glucagon-like peptide-1 receptor agonists in patients with HFpEF. The STEP-HFpEF and STEP-HFpEF DM trials showed that weekly semaglutide injections significantly improved exercise capacity, reduced HF symptoms, and enhanced physical function in patients with HFpEF, particularly those with obesity and diabetes [99, 100]. These benefits may stem, in part, from significant weight loss, which reduces the hemodynamic burden on the heart and provides potent anti-inflammatory effects that counteract systemic inflammation [99]. However, the associated weight reduction, comprising approximately 20% to 50% muscle loss, underscores the importance of integrating structured exercise programs to mitigate muscle loss while augmenting cardiovascular and functional gains [101].

Dietary modifications enhance the efficacy of exercise interventions, particularly in patients with HFpEF and obesity. Caloric restriction combined with exercise has shown significant additive effects resulting in a large 2.5 mL/kg/min increase in peak VO_2_, approximately double the improvement observed with either intervention alone, along with a 10% weight loss and reduced systemic inflammation [25]. While whey protein and vitamin D supplementation may support muscle synthesis, they have not yet been proven to enhance exercise training outcomes in HFpEF [5]. Low-sodium and DASH diets may improve exercise tolerance in patients with HFpEF and hypertension through favorable changes in diastolic function, arterial elastance, and ventricular–arterial coupling [102]. A Mediterranean-style diet, rich in unsaturated fatty acids, also shows promise in improving cardiorespiratory fitness in patients with HFpEF and obesity, though more robust evidence from RCTs is needed [5, 103]. Emerging approaches, such as ketogenic diets, may offer metabolic advantages for specific HFpEF populations but require further study [104].

## Future directions

### Patient selection and advanced phenotyping

The heterogeneity of HFpEF presents significant challenges in optimizing exercise interventions. While HFpEF disproportionally affects older adults and women, its clinical presentation is highly variable [2, 82, 105, 106]. Obesity, for instance, worsens symptoms through increased left ventricular stiffness and systemic inflammation, further impairing functional capacity [14, 67]. Similarly, AF reduces cardiac output during exertion by impairing ventricular filling, compounding exercise intolerance [105]. Despite these observations, traditional clinical phenotyping, such as by body mass index and AF, has not reliably determined differential response rates [107, 108]. Addressing this complexity may require moving beyond baseline clinical characteristics toward integrative profiling that combines physiologic, functional, and molecular insights to better predict and optimize exercise responses in HFpEF.

Future approaches to exercise training in HFpEF may follow two parallel, complementary paths forward. The first involves advanced comprehensive phenotyping integrating layers of assessment to identify physiologic and molecular mechanisms driving individual exercise responses. Understanding the relative impact of central and peripheral factors, such as ventricular-arterial coupling, skeletal muscle dysfunction, frailty, and cardio-metabolic inflammation, may provide insights to guide exercise training. For instance, muscle mass and strength assessments can guide the incorporation of resistance training to mitigate sarcopenia and improve overall functional capacity [40]. For frail older patients with HFpEF, the Clinical Frailty Scale can inform the safe progression of exercise intensity, starting with lower-intensity continuous or multi-modal training and gradually increasing as tolerated [109]. Molecular profiling of biomarkers reflecting systemic inflammation, oxidative stress, and metabolic dysfunction may help identify patients who could benefit from interventions like caloric restriction for weight loss in combination with exercise. Cardiovascular proteomics linked to key exercise phenotypes, such as reduced peak VO_2_ and impaired oxygen extraction capacity, could potentially guide targeted exercise regimens to improve these specific limitations [110]. These insights from comprehensive phenotyping could inform individualized exercise prescriptions that address the unique pathophysiologic drivers of exercise intolerance in each patient with HFpEF.

The second approach focuses on implementing proven, standardized regimens, such as MICT, HIIT, and resistance training (Table 2), to deliver immediate and scalable median benefits for the HFpEF population. While less precise than tailored interventions, standardized protocols provide a pragmatic solution, particularly in resource-limited settings without access to advanced phenotyping tools. Regardless of the approach, safety is paramount. For example, initial supervised sessions with electrocardiographic monitoring, particularly for patients with AF or CAD, can help detect arrhythmias or ischemia during exertion [15]. In turn, exercise intensity can be gradually increased to build tolerance without exacerbating symptoms and preventing musculoskeletal injuries.

### Research gaps and future priorities

Despite growing evidence supporting exercise as a beneficial intervention in HFpEF, significant knowledge gaps persist. The optimal type, intensity, duration, and frequency of exercise to achieve sustained, long-term benefits remain limited in definition. While prior studies described in this review provide distinct benefits, it is unclear how to best combine or tailor interventions based on the heterogeneity of HFpEF. Most studies focus on short-term metrics (e.g., exercise capacity and QoL), leaving the long-term impact of exercise interventions on clinical outcomes like hospitalizations, mortality, and healthcare costs understudied.

To address these knowledge gaps, future research should prioritize large-scale, multicenter randomized controlled trials evaluating the effects of exercise interventions on long-term outcomes in HFpEF. The ongoing REHAB-HFpEF trial represents an important step in this direction, evaluating a tailored physical rehabilitation program for older patients hospitalized with acute decompensated HFpEF, with a focus on outcomes including all-cause rehospitalizations, mortality, and mobility-related disability [59].

Additional research priorities include developing tailored exercise programs for patient-specific HFpEF phenotypes and investigating the synergistic effects of combining exercise and nutritional interventions with pharmacological treatments, particularly given the expanding range of therapeutic options. The potential of wearable devices and telemedicine platforms to enhance exercise training through remote monitoring and personalized feedback warrants rigorous investigation, especially given their ability to improve access for patients in remote areas and facilitate real-time outcome tracking. Further research into the cost-effectiveness of exercise interventions, especially when integrated with pharmacologic therapies, is essential to support widespread clinical adoption. Lastly, the role of exercise in preventing HFpEF in at-risk populations (i.e., stage A/B HF) also represents a critical gap in evidence that requires exploration.

## Conclusions

Exercise interventions are a proven therapy for patients with HFpEF, demonstrating significant benefits in exercise capacity, physical function, and QoL. Current evidence most supports aerobic/endurance and resistance exercise as effective regimens for improving outcomes. By addressing remaining gaps in evidence, the field can move toward more comprehensive and individualized care for patients with HFpEF to improve the durability of exercise capacity outcomes and clinical endpoints.

## Supplementary Information

Below is the link to the electronic supplementary material.

Supplementary file1 (DOCX 52 KB)

## Data Availability

No datasets were generated or analysed during the current study.
