# Supplementary material for: Aerobic, resistance, and specialized exercise training in heart failure with preserved ejection fraction: A state-of-the-art review
Source: Heart Fail Rev. 2025 May 15;30(5):1015–34. doi: 10.1007/s10741-025-10526-x (PMC12296771; doi:10.1007/s10741-025-10526-x)
Supplement: Supplementary file 1 — Supplementary file1 (DOCX 52 KB) [file 10741_2025_10526_MOESM1_ESM.docx]

**SUPPLEMENTAL APPENDIX**

**Aerobic, Resistance, and Specialized Exercise Training in Heart Failure with Preserved Ejection Fraction: A State-of-the-Art Review**

**Running head**: Exercise Training in HFpEF

Saeid Mirzai, DO, MS;^1^ Uttsav Sandesara, DO;^1^ Mark J. Haykowsky, PhD;^2^ Peter H. Brubaker, PhD;^3^ Dalane W. Kitzman, MD;^1,4^ Anthony E. Peters, MD, MS^1^

^1^ Section on Cardiovascular Medicine, Department of Internal Medicine, Wake Forest University School of Medicine, Winston-Salem, NC, USA

^2^ Integrated Cardiovascular Exercise Physiology and Rehabilitation Lab, Faculty of Nursing, College of Health Sciences, University of Alberta, Edmonton, Alberta, Canada

^3^ Department of Health and Exercise Science, Wake Forest University, Winston Salem, NC, USA

^4^ Section on Gerontology and Geriatric Medicine, Department of Internal Medicine, Wake Forest University School of Medicine, Winston Salem, NC, USA

**TEXT**

None.

**TABLES**

| **Table S1**. Study population characteristics from randomized trials evaluating exercise interventions in heart failure with preserved ejection fraction. | | | | | | | | |
| --- | --- | --- | --- | --- | --- | --- | --- | --- |
| **Year** | **First author** | **Country** | **HFpEF definition** | **Age (years)** | **Sex (% female)** | **Race/ethnicity (% white)** | **Mean BMI (kg/m^2^)** | **NYHA class** |
| 2004 | Gary | USA | ≥45% | 67±11 (ET), 69±11 (control) | 100% | 63% (ET), 56% (control) | 35±6 (ET), 32±7 (control) | II-III |
| 2010 | Kitzman (PARIS 1) | USA | ≥50% | 70±6 (ET), 69±5 (control) | 83% (ET), 91% (control) | 88% (ET), 73% (control) | 30±6 (ET), 31±7 (control) | II-III |
| 2011 | Edelmann (Ex-DHF Pilot) | Germany | ≥50% | 64±8 (ET), 65±6 (control) | 55% (ET), 60% (control) | NR | 31±6 (ET), 31±4 (control) | II-III |
| 2012 | Alves | Israel | ≥55% | 62.9±10.2 | 29.0% | NR | 28.4±4.5 | I-III |
| 2012 | Smart | Australia | >45% | 67±5.8 (ET), 61.9±6.9 (control) | 100% | NR | 31.1±5.5 (ET), 33.1±7.3 (control) | I-II |
| 2013 | Karavidas | Greece | >50% | 69.4±8.6 (ET), 68.5±7.9 (control) | 60% in both groups | NR | NR | II-III |
| 2013 | Kitzman (PARIS 2) | USA | ≥50% | 70±7 (ET), 70±7 (control) | 72% (ET), 80% (control) | 66% (ET), 71% (control) | 32.2±6.7 (ET), 32.0±6.6 (control) | II-III |
| 2013 | Yeh | USA | ≥50% | 68±11 (tai chi), 63±11 (AT) | 50% in both groups | 100% (tai chi), 62% (AT) | 32±10 (tai chi), 34±14 (AT) | I-III |
| 2014 | Palau | Spain | >50% | 68 (60-76) (ET), 74 (73-77) (control) | 50% in both groups | NR | 34.3 (28.2-38) (ET), 30 (26-32) (control) | II-III |
| 2015 | Angadi | USA | Not reported | 69.0±6.1 (HIIT), 71.5±11.7 (MICT) | 11% (HIIT), 33% (MICT) | NR | 29.8±5.1 (HIIT), 29.3±2.8 (MICT) | II-III |
| 2016 | Fu* | Taiwan | ≥50% | 60.5±2.7 (HFpEF ET), 63.1±2.6 (HFpEF control) | 33% (HFpEF ET), 40% (HFpEF control) | NR | NR | II-III |
| 2016 | Kitzman (SECRET 1) | USA | ≥50% | 67±5 | 81% | 55% | 39.3±5.6 | II-III |
| 2017 | Shaltout | USA | ≥50% | 68.0±6.2 (ET+BRJ), 70.6±7.6 (ET+placebo) | 80% (ET+BRJ), 89% (ET+placebo) | 60% (ET+BRJ), 67% (ET+placebo) | 33.5±5.8 (ET+BRJ), 31.5±5.4 (ET+placebo) | II-III |
| 2018 | Lang (REACH-HF) | Scotland | ≥45% | 71.8±9.9 (ET), 76.0±6.6 (control) | 64% (ET), 44% (control) | 100% | 32.1±6.3 (ET), 32.2±5.3 (control) | I-III |
| 2019 | Palau (TRAINING-HF) | Spain | >50% | 75±9 (UC), 75±10 (IMT), 72±9 (FES), 73±10 (IMT+FES) | 69.2% (UC), 53.3% (IMT), 60.0% (FES), 50.0% (IMT+FES) | NR | 34.8±5.4 (UC), 30.5±4.3 (IMT), 31.5±4.4 (FES), 31.6±5.9 (IMT+FES) | II-III |
| 2020 | Azhar | USA | >50% | 71±2.8 (Protein+ET), 67±3.9 (Protein only), 72±3.5 (control) | 60% (Protein+ET), 40% (Protein only), 50% (control) | 80% (Protein+ET), 20% (Protein only), 100% (control) | NR | NR |
| 2020 | Donelli da Silveira | Brazil | >50% | 60±10 (HIIT), 60±9 (MICT) | 70% (HIIT), 56% (MICT) | NR | 33±5 (HIIT), 34±6 (MICT) | II-III |
| 2020 | Kinugasa | Japan | ≥45% | 76±7 | 15% | NR | NR | NR |
| 2021 | Kitzman (REHAB-HF)* | USA | ≥45% | 72.7±8.5 (ET), 72.4±7.8 (control) | 58% (ET), 63% (control) | 56% (ET), 54% (control) | 35.4±8.3 (ET), 34.7±9.3 (control) | II-IV |
| 2021 | Mueller (OptimEx-Clin) | Germany, Belgium, Norway | ≥50% | 70±7 (HIIT), 70±8 (MICT), 69±10 (control) | 71% (HIIT), 60% (MICT), 68% (control) | NR | 30.0±5.7 (HIIT), 31.1±6.2 (MICT), 29.0±4.7 (control) | II-III |
| 2022 | Alonso (HEART camp) | USA | ≥50% | 63.3±9.4 (ET), 65.6±9.3 (control) | 44.0% (ET), 47.1% (control) | 56.0% (ET), 52.9% (control) | 35.6±7.2 (ET), 36.4±8.0 (control) | I-III |
| 2023 | Brubaker (SECRET 2) | USA | ≥50% | 69.7±5.8 (RT+CR+AT), 67.9±5.4 (CR+AT) | 86% (RT+CR+AT), 84% (CR+AT) | 48% (RT+CR+AT), 46% (CR+AT) | 39.2±5.6 (RT+CR+AT), 40.0±5.9 (CR+AT) | II-III |
| 2023 | Liu | China | ≥50% | 66.8±6.5 (ET+pill), 64.9±7.6 (ET only), 68.0±4.6 (control) | 44.4% (ET+pill), 29.4% (ET only), 40.0% (control) | NR | 25.6±2.9 (ET+pill), 25.7±3.5 (ET only), 24.5±3.9 (control) | II-III |
| 2024 | Borlaug (INABLE-training) | USA | ≥50% | 72.0 (64.9-76.0) (ET+nitrite), 73.5 (68.7-76.5) (ET+placebo) | 37.8% (ET+nitrite), 55.6% (ET+placebo) | NR | 32.8 (30.8-38.9) (ET+nitrite), 31.6 (27.8-36.7) (ET+placebo) | II-IV |
| 2024 | Obaya | Egypt | >50% | 54.3±2.5 (lower limb), 55.1±2.1 (upper limb) | 0% | NR | NR | NR |
| 2024 | Sharif | USA | >45% | 70±7 (ET) vs 68±7 (control) | 11% (ET), 0% (control) | 100% | 29±3 (ET), 30±5 (control) | I-III |
| 2025 | Edelmann (Ex-DHF) | Germany, Austria | ≥50% | 69.1±7.4 (ET), 70.1±7.1 (control) | 62.1% (ET), 57.1% (control) | 99% | 29.6±5.4 (ET), 29.6±5.1 (control) | II-III |
| **Abbreviations**: AT, aerobic training; BMI, body mass index; BRJ, beetroot juice; CR, caloric restriction; ET, exercise training; FES, functional electrical stimulation; HFpEF, heart failure with preserved ejection fraction; HIIT, high-intensity interval training; IMT, inspiratory muscle training; MICT, moderate-intensity continuous training; NR, not reported; NYHA, New York Heart Association functional classification; RT, resistance training; UC, usual care.  *Data from HFpEF subset; HFrEF excluded. | | | | | | | | |

**FIGURES**

**Figure S1**. PRISMA flow diagram.

**Identification of studies via databases and registers**

Records removed *before screening*:

Duplicate records removed

(n = 415)

Records identified from MEDLINE/PubMed, Embase, and Cochrane databases

(n = 2,122)

**Identification**

Records screened

(n = 1,707)

Records excluded

(n = 1,679)

Reports sought for retrieval

(n = 28)

Reports not retrieved

(n = 0)

**Screening**

Reports excluded:

(n = 9)

Reports assessed for eligibility

(n = 28)

Reports identified from citation searching and assessed for eligibility (n = 8)

Studies included in review

(n = 27)

**Included**
